# Epidemic Model with Isolation in Multilayer Networks

**DOI:** 10.1038/srep12151

**Published:** 2015-07-15

**Authors:** L. G. Alvarez Zuzek, H. E. Stanley, L. A. Braunstein

**Affiliations:** 1 Departamento de Física, Facultad de Ciencias Exactas y Naturales, Universidad Nacional de Mar del Plata, Instituto de Investigaciones Físicas de Mar del Plata (IFIMAR-CONICET), Deán Funes 3350, 7600 Mar del Plata, Argentina; 2 Center for Polymer Studies, Boston University, Boston, Massachusetts 02215, USA

## Abstract

The Susceptible-Infected-Recovered (SIR) model has successfully mimicked the propagation of such airborne diseases as influenza A (H1N1). Although the SIR model has recently been studied in a multilayer networks configuration, in almost all the research the isolation of infected individuals is disregarded. Hence we focus our study in an epidemic model in a two-layer network, and we use an isolation parameter *w* to measure the effect of quarantining infected individuals from both layers during an isolation period *t*_*w*_. We call this process the Susceptible-Infected-Isolated-Recovered (SI_I_R) model. Using the framework of link percolation we find that isolation increases the critical epidemic threshold of the disease because the time in which infection can spread is reduced. In this scenario we find that this threshold increases with *w* and *t*_*w*_. When the isolation period is maximum there is a critical threshold for *w* above which the disease never becomes an epidemic. We simulate the process and find an excellent agreement with the theoretical results.

Most real-world systems can be modeled as complex networks in which nodes represent such entities as individuals, companies, or computers and links represent the interactions between them. In recent decades researchers have focused on the topology of these networks^1^. Most recently this focus has been on the processes that spread across networks, e.g., synchronization^2^,^3^, diffusion^4^, percolation^5^,^6^,^7^,^8^, or the propagation of epidemics^9^,^10^,^11^,^12^,^13^,^14^,^15^,^16^,^17^. Epidemic spreading models have been particularly successfully in explaining the propagation of diseases and thereby have allowed the development of mitigation strategies for decreasing the impact of diseases on healthy populations.

A commonly-used model for reproducing disease spreading dynamics in networks is the susceptible-infected-recovered (SIR) model^18^,^19^. It has been used to model such diseases as seasonal influenza, such as the SARS^20^. This model groups the population of individuals to be studied into three compartments according to their state: the susceptible (S), the infected (I), and the recovered (R). When a susceptible node comes in contact with an infected node it becomes infected with an intrinsic probability *β* and after a period of time *t*_*r*_ it recovers and becomes immune. When the parameters *β* and *t*_*r*_ are made constant, the effective probability of infection is given by the transmissibility 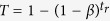
5,21.

As infected individuals cannot be reinfected, the SIR model has a tree-like structure with branches of infection that develop and expand. Because in its final state this process can be mapped into link percolation^7^,^22^, we use a generating function to describe it. In this framework, the most important magnitude is the probability *f* that a branch of infection will expand throughout the network^1^,^22^. When a branch of infection reaches a node with *k* connections across one of its links, it can only expand through its *k* − 1 remaining connections. It can be shown that *f* verifies the self-consistent equation *f* = 1 − *G*_1_(1 − *Tf*), where 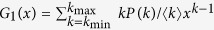 is the generating function of the underlying branching process^7^. Note that *G*_1_(*x*) here represents the probability that the branches of infection will not expand throughout the network. At the final state of this process, the branches of infection contribute to a spanning cluster of recovered, previously infected individuals. Thus the probability of selecting a random node that belongs to the spanning cluster is given by *R* = 1 − *G*_0_(1 − *Tf*), where 
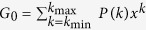
 is the generating function of the degree distribution. When *T* ≤ *T*_*c*_ there is an epidemic-free phase with only small outbreaks, which correspond to finite cluster in link percolation theory. But, when *T* > *T*_*c*_ an epidemic phase develops. In isolated networks the epidemic threshold is given by *T*_*c*_ = 1/(*κ* − 1), where *κ* is the branching factor that is a measure of the heterogeneity of the network. The branching factor is defined as *κ* ≡ 〈*k*^2^〉/〈*k*〉, where 〈*k*^2^〉 and 〈*k*〉 are the second and first moment of the degree distribution, respectively.

Because real-world networks are not isolated, in recent years scientific researchers have focused their attention on multilayer networks, i.e., on “networks of networks”^23^,^24^,^25^,^26^,^27^,^28^,^29^,^30^,^31^,^32^,^33^,^34^,^35^,^36^. In multilayer networks, individuals can be actors on different layers with different contacts in each layer. This is not necessarily the case in interacting networks. Dickinson *et al.*^37^ studied numerically the SIR model in two networks that interact through inter-layer connections given by a degree distribution. There is a probability that these inter-layer connections will allow infection to spread between nodes in different layers. They found that, depending on the average degree of the inter-layer connections, one layer can be in an epidemic-free phase and the other in an epidemic phase. Yagan *et al.*^38^ studied the SIR model in two multilayer networks in which all the individuals act in both layers. In their model the transmissibility is different in each network because one represents the virtual contact network and the other the real contact network. They found that the multilayer structure and the presence of the actors in both layers make the propagation process more efficient and thus increase the theoretical risk of infection above that found in isolated networks. This can have catastrophic consequences for the healthy population. Sanz *et al.*^16^ studied the spreading dynamics and the temporal evolution of two concurrent diseases that interact with each other in a two-layer network system, for different epidemic models. In particular, they found that for the SIR in the final state this interaction can determinate the values of the epidemic threshold of one of the diseases whose dynamic has been modified by the presence of the other disease. Buono *et al.*^39^ studied the SIR model, with *β* and *t*_*r*_ constant, in a system composed of two overlapping layers in which only a fraction *q* of individuals can act in both layers. In their model, the two layers represent contact networks in which only the overlapping nodes enable the propagation, and thus the transmissibility *T* is the same in both layers. They found that decreasing the overlap decreases the transmissibility compared to when there is a full overlap (*q* = 1).

All of the above research assumes that individuals, independent of their state, will continue acting in many layers. In a real-world scenario, however, an infected individual may be isolated for a period of time and thus may not be able to act in other layers, e.g., for a period of time they may not be able to go to work or visit friends and may have to stay at home or be hospitalized. Thus the propagation of the disease is reduced. This scenario is more realistic than the one in which an actor continues to participate in all layers irrespective of their state^38^,^39^. As we will demonstrate, with our approach the critical probability of infection is higher than the one produced by the SIR model in a multilayer network.

## Results

### Model and Simulation Results

We consider the case of a two-layer network, *A* and *B*, of equal size *N*, where one layer represents an individual’s work environment and the other their social environment. The degree distribution in each layer is given by *P*_*i*_(*k*), with *i* = *A*, *B* and *k*_min_ ≤ *k* ≤ *k*_max_, where *k*_min_ and *k*_max_ are the minimum and the maximum degree allowed a node.

At the initial stage of the Susceptible-Infected-Isolated-Recovered model (SI_I_R) all individuals in both layers are susceptible nodes. We randomly infect an individual in layer *A*. At the beginning of the propagation process, each infected individual is isolated from both layers with a probability *w* for a period of time *t*_*w*_. For simplicity, in our epidemic model, we assume that every infected individual is isolated from both layers with the same probability *w* during a period of time *t*_*w*_. The probability that an infected individual is not isolated from both layers is thus 1 − *w*. At each time step, a non-isolated infected individual spreads the disease with a probability *β* during a time interval *t*_*r*_ after which he recover. When an isolated individual *j* after *t*_*w*_ time steps is no longer isolated he reverts to two possibles states. When *t*_*w*_ < *t*_*r*_, *j* will be infected in both layers for only *t*_*r*_ − *t*_*w*_ time steps and the infection transmissibility of *j* is reduced from 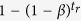 to 
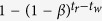
, but when *t*_*w*_ ≥ *t*_*r*_, *j* recovers and no longer spreads the disease. At the final stage of the propagation all of the individuals are either susceptible or recovered. The overall transmissibility 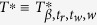 is the probability that an infected individual will transmit the disease to their neighbors. This probability takes into account that the infected is either isolated or non-isolated in both layers for a period of time and is given by


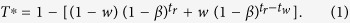


Here the second and third term takes into account non-isolated and isolated individuals and represents the probabilities that this infected individual does not transmit the disease during *t*_*r*_ and *t*_*r*_ − *t*_*w*_ time steps respectively.

**Figure 1:**
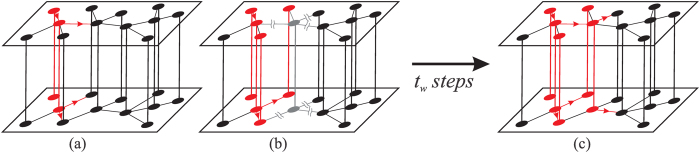
Schematic of a multilayer network consisting of two layers, each of size *N* = 12. The black nodes represent the susceptible individuals and the red nodes the infected individuals. In this case, we consider *t*_*w*_ < *t*_*r*_. (**a**) The red arrows indicate the direction of the branches of infection. All the branches spreads through *A* and *B* because the infected nodes are not isolated and thus interact in both layers. (**b**) The gray node, represents an individual who is isolated from both layers for a period of time *t*_*w*_. (**c**) After *t*_*w*_ time steps the gray node in (**b**) is no longer isolated, and can infect its neighbors in *A* and *B*, if they were not reach by another branch of infection, during *t*_*r*_ − *t*_*w*_ time steps (Color on line).

Mapping this process onto link percolation in two layers, we can write two self-consistent coupled equations, *f*_*i*_, *i* = *A*, *B*, for the probability that in a randomly chosen edge traversed by the disease there will be a node that facilitates an infinite branch of infection throughout the two-layer network, i.e.,


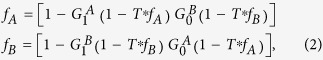


where 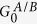 and 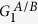 are the generating function defined in the Introduction for layer *A* and *B*. Here 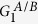 takes into account the probability that a branch of infection reaches a node in layer *A*/*B* of connectivity *k* across one of its links and cannot expand through its remaining *k* − 1 connection. Then 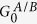 represents the probability that the branch of infection propagates from one layer into the other, reaches a node, but cannot expand through all of its connections. Figure 1 shows a schematic of the contributions to Eqs. (2).

Using the nontrivial roots of Eq. (2) we compute the order parameter of the phase transition, which is the fraction of recovered nodes *R*, where *R* is given by


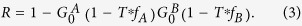


Note that in the final state of the process the fraction of recovered nodes in layers *A* and *B* are equal because all nodes are present in both layers. From Eqs. (1) and (2) we see that if we use the overall transmissibility T^*^ as the control parameter we lose information about *w*, the isolation parameter, and *t*_*w*_, the characteristic time of the isolation. In our model we thus use 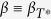 as the control parameter, where *β* is obtained by inverting Eq. (1) with fixed *t*_*r*_. Notice that *β* and *t*_*r*_ are the intrinsic probability of infection and recovery time of an epidemic obtained from epidemic data. Thus making *t*_*r*_ constant means that it is the average time of the duration of the disease.

**Figure 2:**
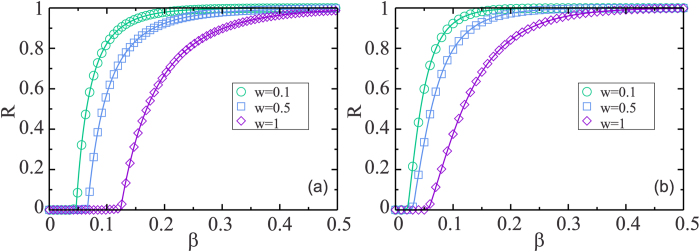
Simulations and theoretical results of the total fraction of recovered nodes *R*, in the final state of the process, as a function of *β*, with *t*_*r*_ = 6 and *t*_*w*_ = 4, for different values of *w*. The full lines corresponds to the theoretical evaluation of Eq. (3) and the symbols corresponds to the simulations results, for *w* = 0.1 (○) (d) in green, *w* = 0.5 (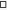) (W) in blue and *w* = 1 (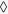) in violet. The multilayer network is consisted by two layers, each of size *N* = 10^5^. For (**a**) two ER layers with 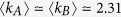, *k*_min_ = 1 and *k*_max_ = 40 and (**b**) two scale free networks with *λ*_*A*_ = 2.5, *λ*_*B*_ = 3.5 and exponential cutoff *c* = 20 with *k*_min_ = 2 and *k*_max_ = 250 (Color online).

Figure 2 shows a plot of the order parameter *R* as a function of *β* for different values of *w*, with *t*_*r*_ = 6 and *t*_*w*_ = 4 obtained from Eq. (3) and from the simulations. For (a) we consider two Erdös-Rényi (ER) networks^40^, which have a Poisson degree distribution and an average degree 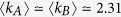, and for (b) we consider two scale free networks with an exponential cutoff *c* = 20^7^, where 
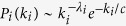
, with *λ*_*A*_ = 2.5 and *λ*_*B*_ = 3.5. We use this type of SF network because it represents many structures found in real-world systems^41^,^42^.

In the simulations we construct two uncorrelated networks of equal size using the Molloy-Reed algorithm^43^, and we randomly overlap one-to-one the nodes in network *A* with the nodes of networks *B*. We assume that an epidemic occurs at each realization if the number of recovered individuals is greater than 200 for a system size of *N* = 10^5^ 44. Realizations with fewer than 200 recovered individuals are considered outbreaks and are disregarded.

**Figure 3:**
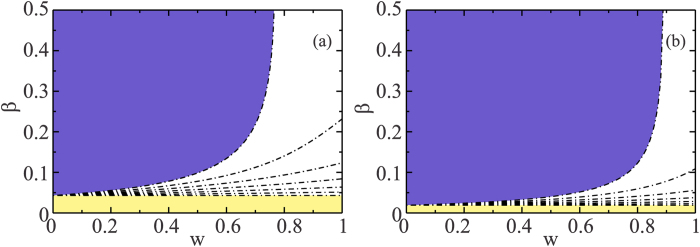
Phase diagram in the plane *β* − *w*. In both plots, we consider *t*_*r*_ = 6 and *t*_*w*_ = 0, 1, 2, 3, 4, 5, 6 from bottom to top for (**a**) two ER networks with 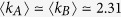 with *k*_min_ = 1 and *k*_max_ = 40. (**b**) two power law networks with *λ*_*A*_ = 2.5 and *λ*_*B*_ = 3.5 with *k*_min_ = 2 and *k*_max_ = 250 and exponential cutoff *c* = 20. The region above each line corresponds to the Epidemic phase and the region below correspond to the Epidemic-free phase. In the limit of *w* → 0 and for *t*_*w*_ = 0 we recover the SIR in multiplex networks with (**a**) *β*_*c*_ ≈ 0.043 and (**b**) *β*_*c*_ ≈ 0.019. For the case *t*_*r*_ = *t*_*w*_, there is a threshold for *w* with (**a**) *w*_*c*_ = 0.76 and (**b**) *w*_*c*_ = 0.88, above which there is only an Epidemic-free phase.

**Figure 4:**
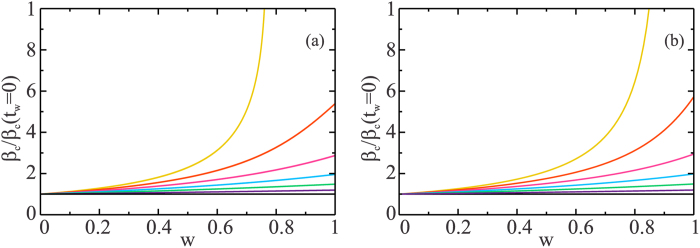
Ratio of *β*_*c*_(*t*_*w*_) to *β*_*c*_(0) as a function of *w*. For *t*_*w*_ = 0, 1, 2, 3, 4, 5, 6 from bottom to top for (**a**) two ER networks with 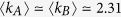 with *k*_min_ = 1 and *k*_max_ = 40 and (**b**) two power law networks with *λ*_*A*_ = 2.5 and *λ*_*B*_ = 3.5 with *k*_min_ = 2 and *k*_max_ = 250, with exponential cutoff *c* = 20. In both Figures, the limit *w* → 0 correspond to a SIR process, and as *w* increases the underestimation increases.

Figure 2 shows an excellent agreement between the theoretical equations (see Eq. (3)) and the simulation results. The plot shows that the critical threshold for an epidemic *β*_*c*_ increases with the isolation parameter *w*. Note that above the threshold but near it *R* decreases as the isolation *w* increases, indicating that isolation for even a brief period of time reduces the propagation of the disease. The critical threshold *β*_*c*_ is at the intersection of the two Eqs. (2) where all branches of infection stop spreading, i.e., *f*_*A*_ = *f*_*B*_ = 0. This is equivalent to finding the solution of the system *det*(*J* − *I*) = 0, where *J* is the Jacobian of the coupled equation with 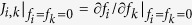 and *I* is the identity, and


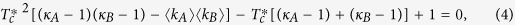


where *κ*_*A*_ and *κ*_*B*_ are the branching factor of layers *A* and *B*, and 〈*k*_*A*_〉 and 〈*k*_*B*_〉 are their average degree. Using numerical evaluations of the roots of Eq. (4) we find the physical and stable solution for the critical threshold *β*_*c*_, which corresponds to the smaller root of Eq. (4)^45^. Figure 3 shows a plot of the phase diagram in the plane *β* − *w* for (a) two ER multilayer networks^40^ with average degree 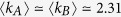 and (b) two power law networks with an exponential cutoff *c* = 20^7^, with *λ*_*A*_ = 2.5 and *λ*_*B*_ = 3.5. In both Figs 3 and 4 we use *t*_*r*_ = 6 and values *t*_*w*_ = 0, 1, 2, 3, 4, 5, and 6, from bottom to top.

The regions below the curves shown in Fig. 3 correspond to the epidemic-free phase. Note that for different values of *t*_*w*_ those regions widen as *w* increases. Note also that when *t*_*r*_ = *t*_*w*_ there is a threshold *w*_*c*_ above which, irrespective of the critical epidemic threshold (*β*_*c*_), the disease never becomes an epidemic. For *t*_*w*_ = 0 and *w* = 0 we recover the SIR process in a two-layer network system that corresponds to *β*_*c*_ ≈ 0.043 with *k*_min_ = 1 and *k*_max_ = 40 in Fig. 3(a) and *β*_*c*_ ≈ 0.019 with *k*_min_ = 2 and *k*_max_ = 250 in Fig. 3(b). Although in the limit *c* → ∞, *β*_*c*_ → 0, most real-world networks are not that heterogeneous and exhibit low values of *c*^9^,^41^.

As expected and confirmed by our model, the best way to stop the propagation of a disease before it becomes an epidemic is to isolate the infected individuals in both layers until they recover, which corresponds to *t*_*w*_ = *t*_*r*_ and *w* > 0. Because this is strongly dependent upon the resources of the location from which the disease begins to spread and on each infected patient’s knowledge of the consequences of being in contact with healthy individuals, the isolation procedure can be difficult to implement.

We also study a case in which there is isolation in only one layer (for a detailed description see Supplementary Information). We find that there is no critical value *w*_*c*_ above which the phase is epidemic-free, i.e., above *β*_*c*_ and for all values of *w* the disease always becomes an epidemic.

The phase diagram indicates that when the SIR model is applied to multilayer networks, which corresponds to the case *t*_*w*_ = 0, it underestimates the critical threshold *β*_*c*_ of an epidemic. This underestimation can strongly affect the spreading dynamics. Figure 4(a) plots the ratio *β*_*c*_/*β*_*c*_(*t*_*w*_ = 0) as a function of *w* for different values of *t*_*w*_, with *t*_*w*_ > 0 for two ER networks. Figure 4(b) shows how much more the critical threshold is underestimated in the SIR model of two-layer SF networks than in the SI_I_R model.

In the limit *t*_*w*_ = 0 and *w* → 0 we revert to the SIR model in multilayer networks^39^. As *w* increases and when *t*_*w*_ ≠ 0 there is always an underestimation of the critical threshold. Note that when *t*_*w*_ = *t*_*r*_ the plot shows that when the percentage of infected individuals who are hospitalized or isolated in their homes is approximately 40%, for two ER, and 50%, for two SF, the SI_I_R model indicates double the actual critical threshold of infection than that indicated in the SIR model. The declaration of an epidemic by a government health service is a non-trivial decision, and can cause panic and negatively effect the economy of the region. Thus any epidemic model of airborne diseases that spread in multilayer networks, if the projected scenario is to be realistic and in agreement with the available real data, must take into account that some infected individuals will be isolated for a period of time. Note that isolation can represent behavioral change but, unlike previous models in which the behavioral changes are solely the result of decisions made by susceptible individuals^46^,^47^, our model allows behavioral changes brought about by placing the infected individuals in quarantine or by hospitalizing them^48^,^49^,^50^,^51^, two practices that were instituted during the recent Ebola outbreak in West Africa. Also note that this isolation can delay the onset of the peak of the epidemic and thus allow health authorities more time to make interventions. This is an important topic for future investigation.

## Discussion

In summary, we study a SI_I_R epidemic model in a two-layer network in which infected individuals are isolated from both layers with probability *w* during a period of time *t*_*w*_. Using the framework of link percolation based on a generating function, we compute the total fraction of recovered nodes in the steady state as a function of the probability of infection *β* and find a perfect agreement between the theoretical and the simulation results. We derive an expression for the intrinsic epidemic threshold and we find that *β*_*c*_ increases as *w* and *t*_*w*_ increase. For *t*_*w*_ = *t*_*r*_ we find a critical threshold *w*_*c*_ above which any disease never becomes an epidemic and which cannot be found when isolating only in one layer. From our results we also note that as the isolation parameter and the period of isolation increases the underestimation increases. Our model enables us to conclude that the SIR model of multilayer networks without isolation underestimates the critical infection threshold. Thus the isolation of the infected individuals, in both layers, for a period of time should be included in future epidemic models in which individuals can recover.

## Additional Information

**How to cite this article**: Zuzek, L. G. A. *et al.* Epidemic Model with Isolation in Multilayer Networks. *Sci. Rep.* **5**, 12151; doi: 10.1038/srep12151 (2015).

## Supplementary Material

Supplementary Information
