# Supplementary material for: Epidemic Model with Isolation in Multilayer Networks
Source: Sci Rep. 2015 Jul 15;5:12151. doi: 10.1038/srep12151 (PMC4502411; doi:10.1038/srep12151)
Supplement: Supplementary Information [file srep12151-s1.pdf]

Epidemic Model with Isolation in Multilayer Networks

L. G. Alvarez Zuzek,[1,*] H. E. Stanley,[2] and L. A. Braunstein[1,2]

[1] *Departamento de Física, Facultad de Ciencias Exactas y Naturales, Universidad Nacional de Mar del Plata, and Instituto de Investigaciones Físicas de Mar del Plata (IFIMAR-CONICET), Deán Funes 3350, 7600 Mar del Plata, Argentina*

[2] *Center for Polymer Studies, Boston University, Boston, Massachusetts 02215, USA.*

Supplementary Information

We here compare the critical values of our model in which the isolation occurs in both layers, with the critical values obtained by a model in which the isolation occurs in only one layer. This situation could reflect a real-world scenario in which infected individuals do not go to their jobs, in one layer, but still have contact with people, in the other layer. At the initial stage all the N individuals in both layers are susceptible and we randomly infect a node in layer A. With a probability w this node is isolated in layer A, but it is not isolated in layer B and can spread the disease there. During the disease spreading the isolated infected nodes in layer A spread the disease for a shorter period of time than the non-isolated nodes. Thus the transmissibility of isolated individuals in layer A is $1-(1-\beta)^{t_r-t_w}$, and the transmissibility of non-isolated individuals in A and all infected individuals in B is $1-(1-\beta)^{t_r}$. At the final stage of the propagation all individuals are either susceptible or recovered, and the transmissibilities T^A and T^B in layer A and B respectively are

$$
\begin{aligned}
T^A &= 1-\Big[(1-w)\,(1-\beta)^{t_r}+w\,(1-\beta)^{t_r-t_w}\Big],\\
T^B &= 1-\Big[(1-\beta)^{t_r}\Big],
\end{aligned} \tag{S1}
$$

where $(1-w)\,(1-\beta)^{t_r}$ is the probability that a non-isolated infected individual will not transmit the disease for a period of time t_r in layer A, $w\,(1-\beta)^{t_r-t_w}$ is the probability that an infected isolated individual in layer A will not transmit the disease during t_r-t_w time steps, and $(1-\beta)^{t_r}$ is the probability that an infected individual will not transmit the disease until they recover after t_r time steps since they were infected.

---

*Electronic address: `lgalvere@mdp.edu.ar`

Using the theoretical arguments presented in Model and Simulation Results, we write two self-consistent coupled equations for the probability that the branches of infection will expand an infinite cluster of recovered individuals at the final stage of the propagation,

$$\begin{aligned} f_A &= [1 - G_1^A\,(1 - T^A f_A)\; G_0^B(1 - T^B f_B)] \\ f_B &= [1 - G_1^B(1 - T^B f_B)\; G_0^A(1 - T^A f_A)]. \end{aligned} \tag{S2}$$

The critical threshold β_c is at the intersection of the two Eqs. (S2) where all branches of infection do not spread, i.e., $f_A = f_B = 0$. This is equivalent to finding the solution of the system $det(J - I) = 0$, where J is the Jacobian of the coupled equation with $J_{i,k}|_{f_i=f_k=0} = \partial f_i/\partial f_k|_{f_i=f_k=0}$ and I is the identity (See Model and Simulation Results in the main text), from where

$$(T_c^A T_c^B)^2\,[(\kappa_A - 1)(\kappa_B - 1) - \langle k_A\rangle\langle k_B\rangle] - T_c^A(\kappa_A - 1) - T_c^B(\kappa_B - 1) + 1 \;=\; 0. \tag{S3}$$

Here κ_A and κ_B are the branching factors of layers A and B respectively, and $\langle k_A\rangle$ and $\langle k_B\rangle$ are their average degree. The physical and stable solution for the critical threshold β_c corresponds to the smaller root of Eq. (S3).

Figure S1 shows a plot of the phase diagram in the plane $\beta - w$ for (a) two ER multilayer networks [1] with average degree $\langle k_A\rangle \simeq \langle k_B\rangle \simeq 2.31$ and (b) two power-law networks with an exponential cutoff $c = 20$ [2], with $\lambda_A = 2.5$ and $\lambda_B = 3.5$. In Fig. S1 we use $t_r = 6$ and values $t_w = 0$, 1, 2, 3, 4, 5, and 6, from bottom to top.

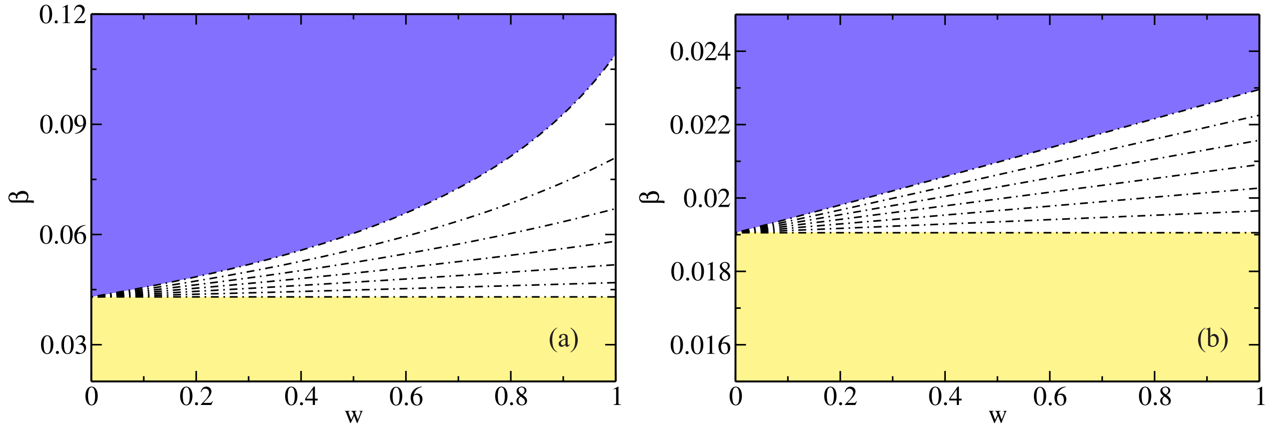

FIG. S1: Phase diagram in the plane $\beta - w$. In both plots, we consider $t_r = 6$ and $t_w = 0, 1, 2, 3, 4, 5, 6$ from bottom to top for (a) two ER networks with $\langle k_A\rangle \simeq \langle k_B\rangle \simeq 2.31$ with $k_{\rm min} = 1$ and $k_{\rm max} = 40$. (b) two power law networks with $\lambda_A = 2.5$ and $\lambda_B = 3.5$ with $k_{\rm min} = 2$ and $k_{\rm max} = 250$ and exponential cutoff $c = 20$. The region above each line corresponds to the Epidemic phase and the region below correspond to the Epidemic-free phase. In the limit of $w \to 0$ and for $t_w = 0$ we recover the SIR in multiplex networks with (a) $\beta_c \approx 0.043$ and (b) $\beta_c \approx 0.019$.

Note that the regions below the curves in Fig. S1 correspond to the epidemic-free phase. For different values of t_w these regions than widen as w increases and reach their maximum size for t_r equal to t_w.

In order to compare the two scenarios in which isolation takes place in both layers or in one layer, in Fig. S2 we plot the phase diagram in the plane $\beta - w$ in both situations for $t_w = 1$, 4, and 6.

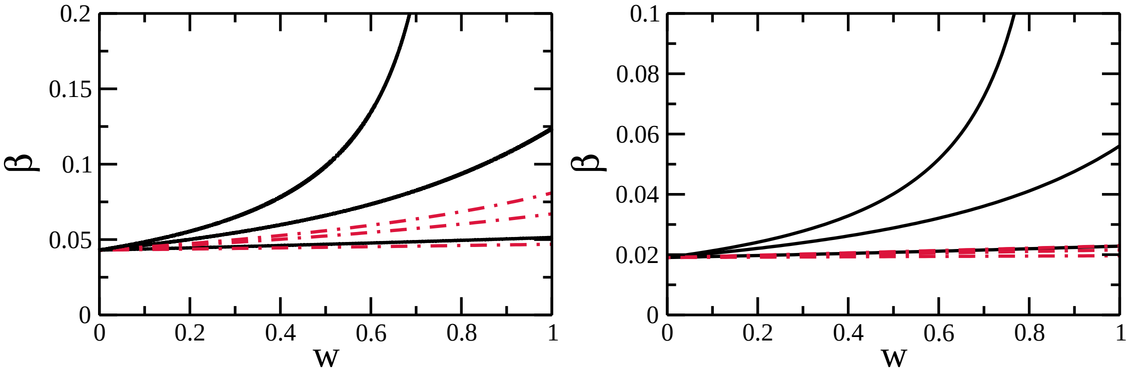

FIG. S2: Phase diagram in the plane $\beta - w$. We consider $t_r = 6$ and $t_w = 1, 4, 6$ from bottom to top. The black lines correspond to isolation in both layers and the red lines correspond to the scenario presented in this section. For (a) two ER networks with $\langle k_A \rangle \simeq \langle k_B \rangle \simeq 2.31$ with $k_{\min} = 1$ and $k_{\max} = 40$. (b) two power law networks with $\lambda_A = 2.5$ and $\lambda_B = 3.5$ with $k_{\min} = 2$ and $k_{\max} = 250$ and exponential cutoff $c = 20$.

As expected in the scenario where isolation takes place in one network, the plot shows that as t_w and w increases the critical values of β increase but much less than in the scenario where isolation is considered in both layers. For example, for two ER networks when $w = 0.5$ and $t_w = 1$ both scenarios have approximately the same value of β, but when $w = 0.5$ and $t_w = 6$ the value of β increases twofold comparing one scenario with the other. When $w > 0.8$, in the scenario presented in this section we find that above some values of β there is an epidemic-phase. In contrast, in the earlier scenario with isolation in both layers there is no value of β above which there is an epidemic-phase, i.e., the spreading disease never becomes an epidemic. When there are two SF layers there is an even more extreme behavior: β varies slightly with w in the scenario with isolation in one layer, but when there is isolation in both layers we find a threshold above which there is no epidemic-phase.

[1] Erdős P. & Rényi A. On Random Graphs. I. *Publications Mathematicae* **6**, 290 (1959).

[2] Newman M. E. J., Strogatz S. H. & Watts D. J. Random graphs with arbitrary degree distributions and their applications. *Phys. Rev. E* **64**, 026118 (2001).
